# Barley in the Production of Cereal-Based Products

**DOI:** 10.3390/plants11243519

**Published:** 2022-12-14

**Authors:** Jasmina Lukinac, Marko Jukić

**Affiliations:** Faculty of Food Technology, JosipJuraj Strossmayer University of Osijek, Franje Kuhača 18, 31 000 Osijek, Croatia

**Keywords:** naked barley, covered barley, cereal-based products, functional food, nutrition value

## Abstract

Barley (*Hordeum vulgare* L.) is unjustly neglected today as a food grain. Interest in the use of barley in the food industry has increased recently. The reason for this is its content of dietary fibre, especially β-glucan, which has been shown to reduce blood cholesterol and lower blood sugar levels. The main nutritional components of barley and barley products, besides the mentioned β-glucan, are starch, sugar, proteins, fat and ash. Although not common in the production of bakery products, barley can be very easily involved in the production of the same products, and such products have improved nutritional characteristics and acceptable sensory characteristics, which make them desirable. Barley has great potential for use in a wide range of cereal-based foods as a partial or full replacement for currently used grains (such as wheat, oats, rice and corn). This article provides basic and general information about the use of barley in food and the processing of barley grains for use in the manufacturing of cereal-based products, with particular attention to the use of barley in the manufacturing of bread (flatbread and leavened bread), noodles and pasta, muffins and cakes and cookies and biscuits.

## 1. Introduction

Barley (*Hordeum vulgare* L.), one of the first domesticated crops, is now one of the most important cereals. It is cultivated worldwide in many countries and regions with temperate climates in summer and some regions with temperate and subtropical climates in winter. Barley was probably first used for human consumption. Later, with the increasing use of wheat in the human diet, barley became important for feed production and an important crop for malt production [1].

Throughout its history, however, it has remained the most important food source for some cultures, especially in Asia and North Africa. Barley has so much to offer as a source of nutrients. Interest in the use of barley in the food industry has increased thanks to its fibre content, especially β-glucan, which has been shown to be effective in lowering blood cholesterol, glycemic index and preventing cardiovascular disease [2].

As it contains soluble dietary fiber, β-glucan and phytochemicals, barley is also called a functional grain. Bread, pasta or biscuits made from barley flour are not yet widely available, but are beginning to occupy an important place in production. However, before it is suitable for human consumption, barley must undergo various stages of processing (such as extrusion, baking, cooking) that significantly affect its composition and physicochemical properties (which play an important role in the development of a new food product) [3].

This article aims to explore the use of barley in the production of cereal-based products. In particular, the use of barley in the production of various cereal-based products such as bread (flatbread and sourdough bread), noodles and pasta, muffins and cakes and cookies and pastries. The first part of the paper describes the historical aspect of barley use, production, taxonomy and anatomy. Other parts of the review deal with the use and application of barley in modern human nutrition and the use of barley in the production of cereal-based products. The results and discoveries of several investigations on these topics are compiled.

## 2. Barley Production and Uses

Archaeological research relating to the lifestyles and history of people in ancient civilizations provides convincing evidence that *Hordeum vulgare,* the subspecies *vulgare* L. (domesticated barley) and *Hordeum spontaneum* C. Koch (wild barley) were the most important foodstuffs, along with pepper, fertile porridge and wheat (*Triticum spelta, Triticum dicoccum* and *Triticum monococcum*) [4]. It is generally believed that modern domesticated or cultivated barley is a direct descendant of wild barley, subspecies *Hordeum vulgare* [5,6].

The theory accepted today is that barley was first tamed in the fertile crescent of the Middle East, which includes present-day Israel, Jordan, Syria and Iran [7,8]. With the intermingling of civilizations and the creation of agricultural trade routes, the use and cultivation of barley spread throughout the European continent. Barley cultivation has moved with the advanced civilizations through Europe and the New World. Historical accounts mention barley as a source of health, strength and stamina for athletes and people doing hard physical labor. Ancient Arabic, Chinese, Egyptian, Ethiopian and Greek literature emphasizes the health benefits and medicinal aspects of barley nutrition. Moreover, the positive properties of barley nutrition are also recognized by more recent civilizations [9].

Barley’s position among the main grains in world production has not changed significantly in the last 15 years. The world production of barley in the crop year 2021/2022 was 147.05 million metric tons (decreasing from around 160.53 million metric tons in 2020/2021), and the European Union was the world’s leading barley producer, with an annual production of 52.75 million metric tons [10]. Much of the world’s barley is produced in regions where cereals such as maize and rice cannot grow well [11]. *Hordeum* species are found in most areas with a Mediterranean climate. The genus is also represented in zones with an oceanic as well as a continental climate [12]. The main reasons for cultivation are the needs of livestock and the increasingly organized and widespread industrial production of cereals for the needs of the brewing industry. For the brewing industry, given the shape of the classes, two-row varieties are the most suitable, while multi-row varieties are commonly used to feed livestock. While barley was grown and used primarily for human food in the last century, it is now widely grown for animal feed, malt products and human nutrition [2]. Barley can be successfully used in many foods at various levels and, in many cases, adds texture, flavor, aroma and nutritional value to the product [13]. Various forms of processed barley grain are used in foods in the form of pearled barley as groats, flakes or flour. In Western countries, pearl barley, whole, flaked or ground, is used in breakfast cereals, stews, soups, porridges, baking flour mixes and baby foods. In Middle Eastern and North African countries, barley is pearled, milled and used in soups, pita bread and porridge [14].

## 3. Types and Forms of Barley

### 3.1. Species and Botanical Characteristics of Barley

Barley belongs to the family *Poaceae* (Gramineae), the division *Triticeae*, the subdivision *Hordeinae*, and the genus *Hordeum* [9]. In addition, barley is classified into different classes based on its different physical and nutritional properties, and its end use is determined depending on which class it belongs to [13]. According to von Bothmer [15], there are 32 species and 45 taxa belonging to *Hordeum*, which are divided into 4 sections, although some authors indicate 6 sections. All cultivated forms of barley are included in one *Hordeum vulgar* species. Members of the genus *Hordeum* are divided into three groups based on the number of chromosomes: diploids (2n = 2x = 14), tetraploids (2n = 4x = 28) and hexploids (2n = 6x = 42). Cultivated barley can be diploid and hexploid, whereas wild barley can only be diploid. Although its origin is not fully understood, modern barley (*H. vulgare*) is thought to be descended from wild barley [9].

The barley grain has a long, twisting appearance. In cross-section, four basic sections of the grain can be distinguished:

- Germ (embryo),
- endosperm,
- aleuron,
- pericarp and testa,
- husk (glume or hull).

Looking at the morphology of barley grains, most of the grain consists of the endosperm, which accounts for 77.2% of the grain weight and is rich in starch and protein [16]. Depending on the barley variety and growing conditions, the husk accounts for an average of 13%; pericarp and testa, 3.3% and aleouron, 5.5%. The germ contains proteins, lipids and tocopherol and accounts for 3% of the barley grain weight [16,17,18].

**Figure 1:**
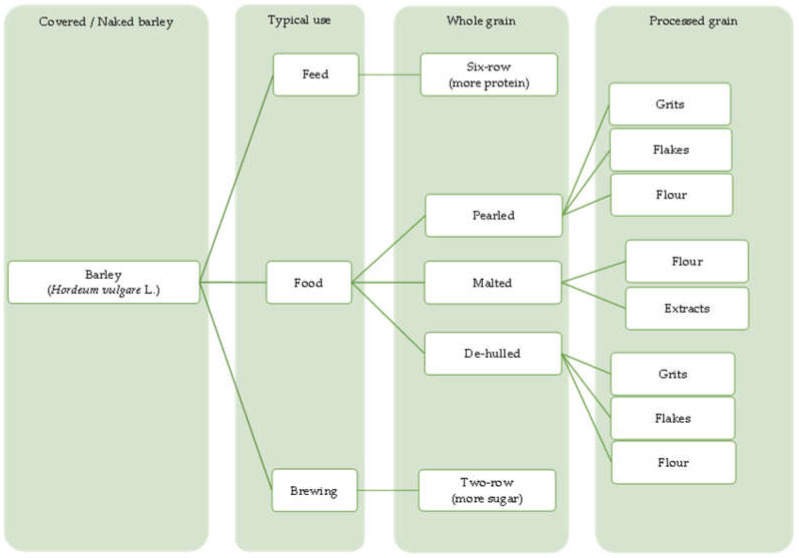
Barley grain classification.

Barley is one of the most genetically diverse cereals. It can be classified as a spring or winter variety, two-rowed or six-rowed, covered or naked/hulless (which refers to the presence or absence of an outer husk on the grain) and brewing, food or feed, which refers to the end use (Figure 1). Depending on the number of rows in the barley heads, there are two main types of barley: six-rowed barley and two-rowed barley. In two-rowed barley, only the middle spikelet is fertile, while in six-rowed barley, the lateral spikelets are also fertile [13,19]. Barley is also referred to as naked (hulless) or covered (characterized by the presence of a glume covering the grain), and as spring or winter barley. Comparing covered and naked barley, naked barley contains more protein, lipids and soluble fiber and, therefore, has better nutritional value than covered barley [20].

Barley is one of the best-adapted cereals grown in climates ranging from subarctic to subtropical. The barley grown is currently used for malt production, and the remainder is used as livestock feed or for human consumption. There are certain barley varieties that are best suited for each of these uses, and some varieties can be used for more than one purpose [21,22].

For human food purposes, barley production is relatively small (~6%) but is becoming increasingly important [23]. For human consumption, barley is used mainly in soups as an additive, and a small portion is milled into flour. Recently, there has been growing interest in the use of certain barley varieties for human nutrition [24]. Among the most valuable barley varieties that positively affect human health are those rich in soluble dietary fiber (such as β-glucan, arabinoxylan or pentosan) [17]. Barley is considered one of the best sources of phytochemicals such as tocols. Tocopherols and tocotrienols (commonly referred to as tocols) are fat-soluble phytochemicals found in barley [25]. Tocols present in the cereal have various health benefits, such as anticancer properties [26], ability to activate immune function [27] and ability to reduce the risk of cardiovascular disease and stroke [28].

However, when we talk about barley varieties and their processing into an edible food source, it is important to distinguish two main types: pearled and naked barley, with pearled barley being the most commonly used. In addition to pearled and naked barley, malt (which is made from barley) is also used in the food industry in baked goods and flavorings [9,19]. Because of its use in malt production, barley is grown in many areas of the world for both cultural and economic reasons. Barley is also grown for feed and was traditionally one of the most important feed grains, which has recently been largely replaced by corn [24].

### 3.2. Processing of Barley Grain

**Figure 2:**
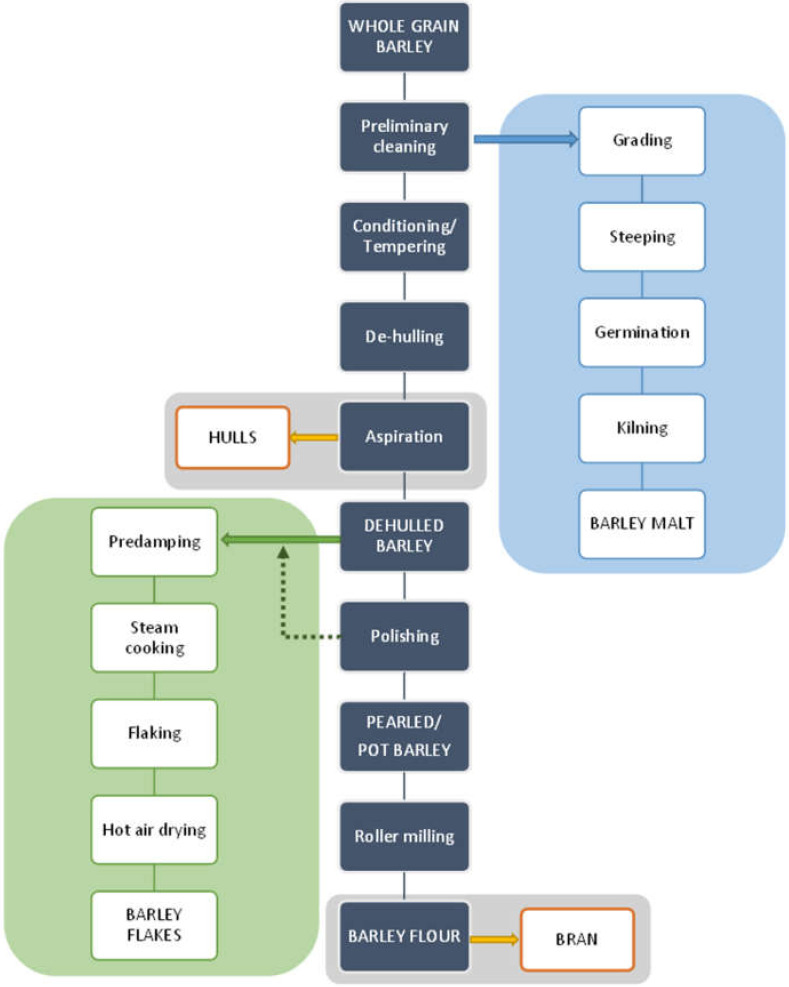
Flow diagram of barley processing [23,29,30].

Barley grain is subject to processing to obtain a barley form suitable for human consumption. The production process of pearled/pot barley, barley flour, barley malt or barley flakes is shown in the flow chart in Figure 2.

In the production process of pearled barley or barley flour by milling, it is first pre-cleaned, then conditioned (or tempered), followed by dehulling and aspiration. The resulting de-hulled barley grains are then fed to the pearling and polishing process, resulting in pearled or pot barley. Finally, barley flour is produced by roller-milling of pearled barley, with barley bran as a by-product of barley milling [23].

In the production of barley flakes, the barley grits are first predamped, then cooked with steam, flaked and finally dried with hot air [29].

Barley malt is the end product of the malting process and is used to make malt-based beverages or ground into flour. After the barley grains have been sorted by sieving, the malting process can begin by steeping the barley in water. This phase is followed by germination, which is responsible for producing the maximum extractable material available. The germination phase is followed by the kilning process (controlled drying of the green malt), which is responsible for developing the malt’s color and flavor properties [30].

### 3.3. Barley Forms and Processing By-Products Available on the Market

There are several forms of barley on the market: whole grain barley, barley processing by-products and barley mill products, which are described below and shown in Table 1.

- *Covered barley* is a grain with an outer inedible husk that should be removed to make the grain suitable for food production (while the bran and endosperm layer may remain intact). It is also known as barley groats. It is rich in fiber, which makes it chewier and take longer to cook. However, naked barley has a greater potential for food development than covered barley. One of the main problems in developing functional barley-based products is that the husk of covered barley must be removed for consumption, which makes covered barley less attractive for food production [31].
- *Roasted Covered Barley*. Roasted barley grains from covered barley are used to prepare non-alcoholic beverages such as coffee substitute and roasted barley tea, called mugi-cha in Japan [32].
- *Barley flakes (rolled barley)* are the result of conditioning, steaming and flaking of pre-cleaned, partially debranned barley grains, flattened and sliced, similar to oat flakes. They are cooked quickly, but have a lower nutritional content than covered barley. Two types of rolled barley flakes can be distinguished: *de-hulled* and *pearled* rolled barley flakes. While de-hulled rolled flakes are made from whole grain barley after de-hulling, pearled rolled flakes are slightly smaller, have an attractive round shape with no discernible bran and can be made from either naked or de-hulled barley [33].
- *Barley malt* is a sprouted grain produced in the malting process by controlled germination of cereal grains that have been previously soaked in water, and then further germination is stopped by drying at high temperatures. Malting involves three main steps: steeping (soaking the barley), germination of grain (to open it up for the fermentation process in which the starch is converted into sugar, which becomes alcohol) and kilning (heating the barley grain to dry it and obtain its final color and flavor) [9]. Barley malt is usually used in the production of beer, whiskey and other alcoholic beverages. Of all the cereals, barley is the most used, but wheat, rye or oats can also be used. The drying temperature affects the type and color of malt, so we distinguish: pale, crystal, coffee, chocolate, black, etc. [34].
- *Malted (sprouted) barley flour* is made from barley malt. Because of its lower gluten content, it is used as a dough conditioner in the production of many products such as bread, pizza crusts, crackers, rolls, pretzels, etc. [9]. It is considered a functional ingredient because it is rich in fiber, especially β-glucan, which can lower cholesterol and blood sugar levels. In addition, malted barley flour is used as an improver in various bakery products [35]. Two types of barley malt flour can be distinguished: diastatic and non-diastatic malt powder.
- *Diastatic malt flour* is neutral in taste and used as an additive for other bread flours with low natural diastatic activity. The addition of diastatic malt flour gives a moister product with a higher protein content [9].
- *Non-diastatic malt barley flour* (malt flour) has no active enzymes in it. It is used for its distinctive flavor. It has many applications, such as in malted milk and in cereal-based products, to give them a softer crumb, glossy surface and complement the taste [36].
- *Naked (hulless) barley* is a barley grain in which the hulled layer falls off naturally and the bran layer and the endosperm layer remain intact during processing. Research confirms the growing interest in its use in manufacturing cereal-based products. The addition of naked barley flour affects the nutritional value of the product by increasing the content of antioxidants and soluble fiber (β-glucan) [36,37,38,39].
- *Pearled (Pearl*) barley is produced by gradually removing the husk, bran and germ by grinding in a stone mill by the process of pearling. It can be produced from covered barley or naked barley. In the case of covered barley, the grain is obtained by removing the husk and germ from the grain. In naked barley, the barrier around the endosperm and germ must be removed to obtain a white grain. This is the most common form of barley. It cooks faster than covered barley but has fewer nutrients.
- In addition, there is another co-variety called *pot barley*. The difference between pot barley and pearled barley is subtle. The production of pot barley occurs in the first stage of pearling (and still has most of the barley bran intact), while further abrasion leads to pearled barley. Pearled barley gets its name from the extra rounds of polishing it undergoes, and is mainly used in traditional recipes as a substitute for rice or as breakfast cereal [2]. The by-products of pearled barley production are used as animal feed. However, recent research suggests that the by-products obtained from pearled barley production are a potential natural source of folate for the manufacturing of cereal-based products [40].
- *Puffed barley* is a type of barley made by puffing naked barley grains at medium temperature and pressure, which does not lose the fiber, vitamins and minerals contained in the grain. Puffed barley has a low glycemic index and is, therefore, suitable for diabetics. In addition, it is known for its anti-inflammatory properties, lowering blood pressure, controlling blood sugar levels by improving insulin response and lowering cholesterol levels. Puffed barley is easily incorporated into various food products or consumed directly [41].
- *Barley sprouts* are obtained from the germination of seeds and their development in water (or other medium), harvested before the development of true leaves and intended for consumption whole (including the seeds). Barley seedlings can be consumed in the form of ready-to-eat sprouts or further processed (dried or roasted) [42]. The use of dried sprouted cereals is possible in producing pasta, noodles, unleavened bread and porridge [43]. Barley sprouts have more available nutrients than mature grains. These nutrients include folic acid, iron, vitamin C, zinc, magnesium and protein [44].
- *Barley brewers’ spent grain* (BSG) is an important by-product of the brewing industry, produced during the malt mashing phase by enzymatic changes that cause about 60–70% of the dry matter to pass into the wort, while the rest remains BSG. It is a lignocellulosic material composed mainly of the husk, germ, protein and non-fermentable fibers, thus having high protein content (20%) and fibers (70%). Its main use was limited to animal feed, energy production and biotechnological processes, although, today, it is used to produce functionally enriched products [45,46].
- *Barley grits* are made from de-hulled or naked barley grains that are roasted and cut into smaller pieces. Sometimes, the pieces are steamed or boiled and then dried for shipment. Steaming or parboiling speeds up the subsequent cooking time and makes the grits last longer. The nutritional content varies depending on the origin (de-hulled or pearled barley) [47].
- *Barley malt* is normally used for brewing and distilling. Malt is a germinated grain that has been dried in the malting process and is used in the production of beer, whiskey and other alcoholic beverages. Malt is produced by controlled germination of cereal grains that have been previously soaked in water, and then further germination is stopped by drying at high temperatures. Of all the cereals, barley is commonly used, but wheat, rye or oats can also be used. The drying temperature affects the type and color of malt, so we distinguish: pale, crystal, coffee, chocolate, black, etc. [34].
- *Barley flour* is usually produced by roller milling of pearled, covered or naked barley. The use of barley flour is varied; it can be used for making wheat-based products (bread, flatbread, cakes, cookies, pasta, extruded snack foods) and for making bread with yeast as a component of composite flours [17]. Because barley flour contains hordeins (not gliadins as in wheat flour), it is less able to form a gluten complex after hydration and mixing.
- *Barley bran* is obtained as a by-product of barley milling naked or de-hulled barley. It consists of testa and pericarp, germ, the three-celled aleurone layer, and the subaleurone layer. Due to its high content of soluble dietary fibre, in particular its β-glucan content, its use is becoming more widespread in baking. The addition of bran to cereal-based products has several effects and increases dough yield. Due to its high water absorption capacity, it causes the formation of a moister and shorter dough, a lower tolerance to fermentation, a lower volume, a tense and inelastic crumb and an altered taste, depending on the type of fiber and the type of bread [48].
- *Barley husk (hull or glume)* usually accounts for about 10–16% of the total dry weight of the grain and consists mainly of arabinoxylan, cellulose, lignin, other phenolic compounds and protein. In naked barley, the husks are removed during threshing, while in covered barley, they remain associated with the pericarp tissue [49]. Barley husk plays no role in the food industry and is classified as waste or production residue. It is a by-product of the food and agricultural industries, obtained by processing barley through milling. Some studies have focused on the use of barley husk as a raw material for producing value-added products, such as ethanol [50]. Cruz et al. [51] investigated the possibilities of chemical-biotechnological processing of barley husk, obtained after the beer brewing process, with sulfuric acid and sodium hydroxide to fully utilize the three fractions cellulose, hemicellulose and lignin and eventually obtain natural lactic acid and phenolic compounds with high antioxidant capacity. Höije et al. [49] analyzed the use of different methods to isolate arabinoxylan from barley husk, the influence of the isolation method on the yield, composition and physicochemical properties of the material. This extraction process yields about 57% of the available arabinoxylan in dry barley husk.
- *Barley starch* is a white to cream-colored powder with a neutral odor and taste [52]. In the dry weight of barley grains, the average starch content varies from 45% (in some Chinese barley varieties [53]) to 72% (in Canadian barley varieties [54]). Genetic variations between different barley varieties are the cause of differences in the starch content, as well as chemical composition, structure and physicochemical properties of barley starch [55]. For example, barley genotypes with a high proportion of high-resistance starch and amylose content usually have a low starch content in the grain [56]. The ratio of amylose, amylopectin and other storage substances affects the physicochemical properties, as well as the final use of starch [57,58]. Based on the concentration of barley amylose, starch can be divided into normal (~25–27% amylose), waxy (not measurable up to <5% amylose) and elevated amylose (>35% amylose) [58,59,60]. It can be used in food (in confectionery, bakery products and snacks, sauces, soups and beer production) and industrial food systems (as a thickener, gelling agent, bulking agent, water binder, colloidal stabilizer, adhesive, texture stabilizer and regulator) [61]. Starch with an amylopectin content of 95 to 100% (waxy starch) is used in the food industry to improve product properties, such as stability, texture, uniformity, increased swelling capacity and water holding capacity, as well as better freeze-thaw ability of foods [62]. Some barley starch products can be used in “gluten-free” and “very low gluten” foods if the remaining gluten content is below the levels set by EU legislation. The physicochemical properties of starch, its functional characteristics and its specific use in food depend on the biological origin of the starch [63]. Depending on the botanical origin, there are differences in the shape, size and composition of starch granules [64]. However, due to low shear strength, heat resistance, thermal degradation and high retrogradation, there are limitations in the use of starch in some industrial food applications. Chemical and physical modifications of starch are made to overcome these shortcomings and meet the properties required by the industry [64], or a native starch blend is used (i.e., starch obtained from various sources) [65]. While modified starches generally exhibit better paste clarity, stability and increased resistance to retrogradation [66], chemical modification of starches is intended to produce thickeners with desired rheological properties during storage and transportation [67].

## 4. Chemical Composition of Barley

The chemical composition of barley can vary greatly due to genotype and environmental conditions during cultivation [12,68].

The main components of barley grain are carbohydrates (starch, sugars and fiber), proteins (amino acids), fat (fatty acids) and ash (minerals), as well as vitamins and phenolic compounds [69]. Oscarson et al. [20] reported the typical chemical composition of covered barley and naked barley (Table 2). Whole grain barley contains between 57.7 and 60.7% starch, 12.2 and 15.1% protein, 2.5 and 2.7% fat, 1.2 and 1.5% sugar, 4.8 and 5.7% β-glucan, 16.6 and 20.6% dietary fiber and 1.6 and 2.1% ash, depending on the variety (covered or naked barley).

It should be emphasized that there is a clear relationship between the chemical composition of barley and its intended use. Thus, barley with high protein content is used for human nutrition or as animal feed. On the other hand, barley with low protein content is used for the production of malt or beer. The importance of the proteins contained in barley is reflected in the improvement of the rheological properties (emulsifying ability and stability, foaming, elasticity, cohesiveness and water retention) of barley-based products.

Due to its chemical composition, barley is considered a functional ingredient in the development of foods with beneficial effects on human health. The positive effect of barley on human health is due to fiber, specifically the presence of β-glucans (in wholegrain barley) [70], tocols and resistant starch. The beneficial effects of β-glucan and tocol from barley are shown to lower serum cholesterol [71] and blood glucose levels [72], while β-glucan and resistant starch lower blood sugar levels and improve intestinal function [73]. In addition, due to the presence of phytochemicals (phenolic acids, flavonoids, lignans, phytosterols and folic acid), barley shows antioxidant and antiproliferative ability and lowers blood cholesterol [55,74,75].

### 4.1. Carbohydrates: Starch and Sugars

Starch is quantitatively the most important component of barley grain, followed by fiber and protein. Starch consists of two structural types: branched glucose chains (amylopectin) and straight-chain glucose chains (amylose), in a 3:1 ratio [76].

Although barley usually contains amylopectin and amylose in a 3:1 ratio, there are also barley varieties whose starch is 95–100% amylopectin, and there are cultivars that contain 40–70% amylose [54] as a percentage of total starch. The term waxy barley is used for varieties with a high amylopectin content, while the term non-waxy barley is used for barley varieties that contain starch with a normal 3:1 ratio of amylopectin to amylose [9].

Waxy barley traditionally contains less total starch than comparable normal species, and lower starch content is usually accompanied by small increases in sugar, sucrose, glucose, fructose and a statistically significant increase in dietary fiber (TDF) in the husk. An increase in the TDF component results in an increase in ß-glucan [77,78]. Small amounts of sugars and oligosaccharides (small polymers) are found in the endosperm. Glucose and fructose (monosaccharides) occur in very small amounts (<0.2%) and are found mainly in the mature endosperm. Small amounts of the disaccharide maltose (0.1 to 0.2%) are sometimes found in the endosperm tissue near the embryo. Sucrose content ranges from 0.74 to 0.84%, 80% of which is found in the embryo [9].

### 4.2. Carbohydrates: Dietary Fiber

The definition of dietary fiber in foods has been controversial for many years, but most experts agree that dietary fiber consists of carbohydrates that cannot be digested by mammalian enzymes. Dietary fiber in barley consists mainly of lignin, cellulose, ß-glucan and arabinoxylan.

#### β-Glucan

Of all the dietary fiber components in barley, β-glucan is probably the most important in terms of human nutrition and health. Although β-glucan content in barley is significantly influenced by environmental factors, researchers generally agree that genetic background is the most important factor in the final β-glucan content of barley [79]. 

β-glucans from different sources consist of glucose molecules that may be linked by specific glycosidic linkage, which consequently affects its physicochemical and biological properties. Cereal β-glucans have β–1,3/1,4 glycosidic linkages without any β–1,6 bonds or branching, whereas non-cereal sources such as yeast and fungi usually have β–1,6 linked branches off the main side chain [80].

Although β-glucans are present in all cereals, their concentration is particularly high in sources such as barley grain (2–20%), oat grain (3–8%) and sorghum grain (1.1–6.2%) [81]. Processed grains, such as de-hulled barley, barley grits, pearl barley and oat flakes, contained 4.83%, 3.96%, 2.95% and 2.55–2.91% β-glucan, respectively [82]. β-glucans form a viscous solution in the digestive tract that slows the absorption of glucose after a meal and helps maintain a good ratio of glucose to insulin in the blood. Since bakery products and breakfast cereals have a high glycemic index, it is recommended to eat cereals such as barley and oats (which have a lower glycemic index and are rich in fiber). Viscous (1,3), (1,4)-β-D-glucans lower blood glucose levels. Therefore, barley can be used as a good source of high-value fibre to reduce the glycemic response in conventional wheat-based foods such as bread [83]. In addition, the European Food Safety Authority (EFSA) and the U.S. Food and Drug Administration (FDA) have approved health claims for barley glucan and soluble fiber from barley in terms of lowering blood cholesterol and reducing the risk of coronary disease [84].

Numerous clinical studies have confirmed the value of barley as a hypocholesterolemic food. Based on these reports, barley foods may be labeled with the health claim that they may reduce the risk of coronary heart disease [85].

### 4.3. Proteins

The protein content of barley is highly variable, ranging from 7 to 25%, according to a large USDA study involving more than 10,000 genotypes [86], although protein content in typical barley tends to be in the 9–13% range [9]. In a study that included 3817 samples of beer barley from western Canada, McMillan and Izydorczyk [87] indicated that the average protein content is 12.2%. Analytical methods to accurately calculate protein content are time-consuming and expensive, whereas the determination of nitrogen is relatively simple, rapid and inexpensive.

Nitrogen content is usually multiplied by 6.25 to estimate protein content because most proteins or protein mixtures contain relatively close to 16.0% nitrogen, i.e., 100/16 = 6.25 [69]. Proteins are classified into four groups according to their solubility:

- Albumins—soluble in water, denatured at elevated temperatures, barley albumin is leucosin;
- globulins—soluble in 5% K_2_SO_4_ solution, partially denatured on heating, barley globulin is edestin;
- prolamins—soluble in 70% ethanol solution, barley prolamin is hordein;
- glutenins—soluble in dilute acids or alkalis, barley glutenin is hordenine [88].

The second classification of barley proteins refers to their biological functions, where we distinguish between seed storage proteins (SSPs) and nonstorage proteins. The most important storage proteins are prolamins, which are called hordeins [89].

### 4.4. Fats

Fats are found throughout the barley grain, with the greatest fat content in the aleurone layer and in the embryo, and account for only 1–2% of the dry weight of the endosperm [90]. Åman et al. [91] reported that they extracted a total fat content of 2.1–3.7% dry matter in 115 barley grains, with an average of 3.0%. The major fatty acids in barley are 23% palmitic acid (16:0), 13% oleic acid (18:1), 56% linoleic acid (18:2) and 8% linolenic acid (18:3) [90,92]. Stearic acid is also present, but in a very small proportion (<1%). In addition to fatty acids, phospholipids are also present [9,92].

### 4.5. Ash

The ash or mineral content of barley ranges from 2.0% to 3.0%, with slightly lower content in naked barley varieties and higher content in covered varieties. The higher content in covered varieties, mainly due to hulls, contains around 6.0% ash, which is 60 to 70% nutritionally inert silicon occurring primarily in the outer lemma. The average amounts of macrominerals (g/100 g) in barley are calcium 0.05, phosphorus 0.35, potassium 0.47, magnesium 0.14, sodium 0.05, 0.14 chlorine, sulfur 0.20 and silicon 0.33. The microminerals (mg/kg) in barley are copper 6.25, iron 45.7, manganese 27.2, zinc 34.4, selenium 0.4 and cobalt 0.07. These data are weighted averages from eight research reports [9].

### 4.6. Vitamins

Whole grains are generally a good source of certain vitamins, especially some of the B-complex vitamins (such as thiamin, riboflavin, niacin, pyridoxine, biotin and folates). The barley caryopsis contains all the vitamins and choline with the exception of vitamins A, D, K, B_12_ and C. The average levels of vitamins in barley grain are presented in Table 3 [9]. Some heat-labile B-complex vitamins may be damaged or destroyed by heat during processing.

## 5. Barley in Modern Human Nutrition

Barley is considered an important food ingredient due to its vital biochemical components (β-glucan, starch, amylose, protein). Furthermore, due to its nutritional composition and its action on various mechanisms, it is known as a food with many beneficial effects on the human body (reduces and prevents cardiovascular diseases and has a positive effect on reducing appetite and obesity, etc.) [93].

Health statistics on chronic diseases and mortality are often associated with the dietary habits of the population. Various health promotion agencies and organizations define the dietary components needed to change people’s health and improve health statistics. Nutritionists advocate dietary changes, such as consuming less saturated and trans fats and more fiber. Barley has attracted the attention of health experts because it contains dietary fiber, particularly β-glucan, which has been shown to lower blood cholesterol levels and induce a lower glycemic response [75,94,95].

In modern times, barley is consumed primarily as an ingredient in soups. To expand the consumption of barley to the majority of the diet, common foods such as bread and pasta that are consumed regularly and frequently must be considered. Wheat is ideal for making these foods, but barley itself is not. Replacing wheat flour with barley flour or fractions of ground barley in leavened bread results in increasing soluble fiber and a decrease in gluten, which, in turn, weakens the cellular structure of the bread. The three obvious effects on bread that occur when white wheat flour is replaced by barley flour are a reduction in bread volume, darker color and lower quality of the crumb. For pasta, changes in color and texture are the most obvious effects. Consumer acceptance of food is strongly influenced by appearance, texture, color and taste. These are all characteristics that can be affected by introducing alternative grains such as barley. Another challenge for product manufacturers is to maintain the beneficial physiological properties of β-glucan during processing and preserve the positive health properties.

Foods containing barley can be considered functional foods, especially after the U.S. Food and Drug Administration granted approval in 2006 [85]. Functional foods are defined as any fresh or processed food with properties to promote health or prevent disease beyond the basic function of providing nutrients. The term was introduced in Japan in the mid-1980s and refers to processed foods that are not only nutritious but also contain ingredients that support specific body functions. Foods made from barley have been classified as functional foods to reduce the risk of cardiovascular disease and modify blood glucose levels to treat and prevent diabetes [96]. Recently, the European Food Safety Authority (EFSA) also published a scientific opinion stating that “the health requirements for β-glucans from oats and barley are justified in terms of maintaining normal blood LDL cholesterol levels, increasing satiety leading to lower energy intake, reducing postprandial glycemic response, and digestive functions” [97].

### 5.1. Application of Barley in the Manufacturing of Cereal-Based Products

The use of barley in the preparation of various types of cereal- and cereal component-based food products is still present today in different cultures around the world, especially in the Middle East, North Africa and Northern and Eastern Europe (Iran, Morocco, Ethiopia, Finland, England, Denmark), Russia and Poland and in Asia (Japan, India, Tibet and Republic of Korea) [98]. Table 4 provides an overview of the various traditional barley-based dishes produced worldwide.

Barley has great potential as an edible grain due to its high nutritional value. It contains low fat, complex carbohydrates for energy, relatively balanced proteins, minerals, vitamins, polyphenols and both insoluble and soluble fiber. Because wheat flour can be partially or fully substituted with barley flour in many unleavened bakery products, there is a trend toward increased use of barley, particularly in whole grain bread, unleavened-type bread and breakfast cereal products.

**Unleavened-type bread (flatbread)**—Flatbreads are a traditional type of bread in many countries [121,122]. There are several types of bread under this common name, such as Turkish balzama and yufka; Indian chapattis, parathas, poori and roti; Jordanian balady (sometimes called pita or pocket bread) and Mexican tortillas.

Fermented cereal-based products require high-quality gluten protein, found only in wheat flour. Therefore, barley flour is mainly used for making unleavened bread (flatbread). When barley flour is used in fermented bakery products, it should not be added in large quantities, and different baking additives should be included in the recipe if it is added in large quantities (e.g., glycerol monostearate in combination with NaCl in chapatti bread) [123].

This bread is very easy to prepare since it is made of several basic ingredients and is characterized by the possibility of making it in different thicknesses (from a few millimeters similar to a tortilla to a few centimeters similar to a balzama). As flatbreads are almost independent of fermentation and their volume is not a decisive quality parameter, it is ideal for the inclusion of barley flour in the recipe.

Many authors have dealt with the production and recipes of flatbread with the addition of barley in different variants such as ground barley flour (barley lines with high protein content [124], naked barley [125] and waxy naked barley [126]), whole barley flour [127] or as an extract of ß-glucan from waxy naked barley [128].

Barley flour was added mainly in combination with wheat flour in the preparation of the different varieties of flatbreads. The proportion of barley flour varied in the samples, indicating the desired characteristics of a particular type of flatbread.

Thus, in the scientific literature, addition of up to 100% barley flour is found in tortilla samples [109,129], 50% in flatbread samples [130], 10–40% in balzami samples [131], 5–100% [124] and 10–50% [101,123,125,132] in chapattis samples and 0–100% in balady samples [127,133].

In addition to the barley flour mentioned above, some studies have investigated the addition of dietary fiber (TDF), such as Hecker et al. [128], who added an extract of β-glucan from waxy naked barley as a dietary fiber supplement to flour tortillas. In addition, Izydorczyk et al. [134] addressed the total soluble fiber content in flatbread samples in their study. Flatbreads that contain up to a 20% high-fiber barley fraction in their recipe positively affect the human body (the amount of dietary fiber increases, and the digestibility of starch is reduced).

The type of flatbread also determines the maximum allowable percentage of barley flour in products such as chapattis and balzama, so, in some cases, up to 30% barley flour is acceptable. So, when the percentage is increased, samples become sensorially unacceptable [125,131].

Considering the health aspect and the benefits for the human body, the content of β-glucan and dietary fiber increases proportionally to the amount of barley flour added. In addition, research confirms that β-glucan in barley with high water absorption capacity is responsible for maintaining the starchy character, thus preserving extensibility during storage [131,135,136,137].

**Leavened-type bread**—Bread is a staple food in all cultures and a convenient product containing health-promoting ingredients such as barley. Barley flour alone is not suitable for making leavened-type bread because it does not have the property of spreading that wheat gluten does, preventing the development of the desired texture and volume of the bread. Adding barley flour or high-fiber milled barley fractions may adversely affect common quality parameters such as color and flavor [9].

Barley flour is less able to form a gluten complex after hydration and mixing because gliadin is replaced by hordenines [138]. Therefore, the addition of barley flour in the bread mixture disturbs the properties of the dough, reduces the volume of the bread [124] and causes the darker color of the bread crumbs, which could be due to weaker properties of barley gluten and the high content of phenolic compounds and fiber.

Niffenegger [139] made yeast bread (and other products) using 100% barley flour, 75% barley flour and 25% wheat flour, 50% barley flour and 50% wheat flour and 100% wheat flour. The volume of bread gradually reduced with the increase in the percentage of barley flour. Sensory tests indicated poor texture and flavor of bread with a higher percentage (75% and 100%) of barley.

The focus of several studies has been to determine the acceptable content of barley flour (in combination with wheat flour) without affecting the quality parameters of bread.

Harlan [140] stated that barley flour might be blended with wheat flour up to 20% without affecting the quality of leavened bread. Harlan further stated that up to 80% barley flour can be included in chemically leavened bread and that in both cases, barley flour darkens the color of the bread and changes the flavor. These facts remain true after more than 80 years, and thus much of the research in recent years has focused on improving the quality of bread with barley flour.

Knuckles et al. [141] made bread to which they added barley flour and β-glucan fractions (obtained by aqueous extraction). Dry-milled and sieved barley flour replaced wheat flour in amounts of 20% and 40%, and fractions of water-extracted barley were added in an amount of 5%. When 40% barley flour was added, all bread quality parameters were low and unacceptable. Bread with 20% dry-milled barley flour and 5% water-extracted fractions was rated acceptable by consumers in terms of appearance, color, texture, taste and odor, despite the smaller volume of the bread and darker color.

Kawka et al. [142] added whole barley flakes to the bread dough in a proportion up to 25%. The addition of barley flakes in a proportion of 15% resulted in a decrease in bread volume, but these samples had the best sensory ratings. In this study, the addition of flakes resulted in satisfactory texture and flavor of the bread and showed that they can be used as an ingredient in many baked goods.

Barley bread can be used to lower the glycemic index of conventional wheat-based foods because barley flour is a rich source of high-quality dietary fiber. Cavallero et al. [83] used barley flour sieved and water-extracted fractions (both enriched with β-glucan) to make bread.

A group of researchers in India [143] added barley flour to bread dough in proportions of 10% and 20%, along with wet gluten and ascorbic acid. For the samples with 20% added barley flour, 15% moist gluten and 20 ppm ascorbic acid were found, and the texture and volume of the bread were similar to the control wheat bread.

The acceptable percentage of added barley flour in the production of leavened bread without affecting its quality (volume, texture, color, porosity) varies up to a maximum of 45%, which is confirmed by many studies [133,135,136,144]. Adding a higher proportion of barley flour results in breads with higher crumb density, higher ash content, greater bread hardness and darker breads with lower protein and gluten content. The substitution of wheat flour with barley flour in the production of bread has a positive effect on the nutritional properties of the product. The partial replacement of wheat flour with barley flour creates a functional and healthy product enriched with dietary fiber, β-glucan and minerals without compromising the organoleptic and nutritional properties [145]. Lin et al. [146] investigated replacing part of the wheat flour with six-row barley flour in the production of steamed bread. Two types of barley flour (barley pearl flour prepared from pearled grain and barley endosperm flour obtained after carefully peeling off all layers around the endosperm and removing the embryo) were used, and the effect of adding barley flour on the dough properties during processing and the quality of the steamed bread as the final product were studied. This study also showed that pearl barley flour contains more dietary fibre. On the other hand, increased amounts of barley flour have a negative effect on the quality of steamed bread (significantly reduces the specific volume, brightness and whiteness index of the bread and causes an increase in hardness and chewiness). Barbari is a traditional Iranian yeast bread made by baking in special ovens. The study conducted by Naji-Tabasi et al. [147] dealt with the production of barbari bread with improved quality and higher nutritional value, made with sourdough and whole wheat and barley flour.

**Noodles and pasta**—Pasta to which barley flour is added is made in various forms and types (such as fettuccine, penne, rigatoni, spaghetti, ziti, etc.). The most common synonyms that can be used to describe these products are macaroni, noodles (traditionally associated with Asian consumers, e. g., Japanese ramen or Chinese Llamian) and spaghetti.

People’s awareness of a healthy diet and the consumption of functional products is leading to an increasing interest in the production of such foods. In line with these trends, there is growing interest in the inclusion of whole grains in the development of new pasta varieties with whole wheat, barley flours [148,149,150], barley milling fractions [151,152,153,154] or with a high fraction of β-glucan from barley [155,156,157]. In this way, pasta is enriched with dietary fiber, β-glucan and minerals, with a darker color than those made from wheat flour, but still with satisfactory organoleptic properties. Recent research is based on the production of pasta from whole grains and high-fiber cereals such as barley (which contains 15.2–16.1% fiber and 4.3–5.0% β-glucan) [158].

The important quality parameters for noodles include a light yellow color of noodles without dark spots, a smooth, non-sticky surface, high strength and elasticity [159,160,161]. To offset the negative effects of barley in pasta production (dark pasta, lower firmness and elasticity, increased losses during cooking, absence of gluten), pasta is made from wheat flour to which a lower percentage of barley flour is added. When investigating the optimal recipe for pasta production, Dexter et al. [153] added barley with different amylose content in their study, and the addition of 20% barley flour proved to be the best in pasta production.

There are studies in which quality barley noodles were produced by adding 100% barley flour combined with the addition of various additives (such as glyceryl monostearate and sodium polyacrylate) that improved the textural properties of the barley noodles [162].

**Muffins and cake**—Muffins and cake products owe their popularity to the practical and easy preparation and durability. The addition of barley to the muffin or cake recipe results in darkening of the sample, lower volume and softer structure. Berglund et al. [163] studied the addition of naked waxy barley milled into whole grain flour and also processed into flakes. In their study, they made a number of products (muffins, cookies and granola bars) with the addition of barley flour in proportions ranging from 26–100%. The products were tested for their physical (such as color, volume and spreading factor) and sensory properties. The results showed adding barley flour led to a reduction in volume, a darker color and a generally better sensory evaluation (in appearance, texture, taste, sweetness and flavor) than wheat flour samples. 

Hudson et al. [164] studied muffins prepared from oat bran (100%), barley bran (40%), and rice bran (60%). Sensory analysis showed acceptable or better overall quality (compared to a commercial muffin made with oat bran). Muffins were also successfully prepared with 100% barley flour, although differences between varieties affected muffin volume and density [68]. 

Newman et al. [165] studied the influence of the addition of naked waxy barley fractions in the production of yeast bread, biscuits, cookies and muffins. The barley fractions differed in the amount of TDF, ranging from 8.8–18.2%. The products were evaluated sensorily, and their volume and dimensions were determined. The yeast bread was the only fiber-enriched product with reduced volume and dimensions. The results of the sensory analysis showed no significant difference between the products with and without the addition of barley. The muffins with the addition of barley were described as “moist”.

**Cookies/biscuits**—When wheat flour is partially or completely replaced by barley flour, the gluten protein of wheat is diluted. Since gluten content is not a determining factor in cookie quality (unlike leavened bread), its absence will not decrease cookie quality (even if they become darker in color and softer in structure). Moreover, adding barley flour makes the cookies a functional product due to the content of phenolic compounds and antioxidant activity, β-glucan and dietary fiber [166]. 

The quality of barley flour in producing cookies is significantly influenced by the genotype and the size of the barley grains. Therefore, when selecting a variety of barley to produce cookies, it is necessary to consider the desired characteristics of the cookies to be obtained.

The results of studies on the effects of adding barley flour to cookies on their quality and nutritional composition (β-glucan) have shown that increasing the content of added barley flour leads to an improvement in flavor, density, color, dryness and graininess, as well as an improvement in the nutritional value [167] of cookies. Frost et al. [168] found that adding barley flour to samples resulted in the enrichment of samples with β-glucan (an addition of 30% barley results in 0.5 g in samples and a 50% addition of β-glucan in samples is 0.8 g). To improve the nutritional, sensory and qualitative properties of cookies, a mixture of several cereals was prepared. It has been confirmed that a mixture of wheat, barley (20%) and buckwheat leads to an increase in iron, calcium and zinc content [93] and fiber, fat, ash and carbohydrate content in cookies [169]. The addition of barley flour affected the darker color of the cookies, greater total phenolics content and antioxidant activity compared to wheat biscuits [166]. The color of the samples varied from pale cream to golden brown and depends on the proportion of barley added [170].

The bran of malted barley is a potentially valuable resource for protein, minerals and dietary fibre (which could be important in the food industry, especially in baking). Therefore, substituting wheat flour with malted barley bran could improve the nutritional quality of baked goods. In a study by Ikuomola et al. [171], the quality of cookies produced from blends of wheat flour and bran of malted barley at different ratios was investigated. The addition of 5% malted barley bran could be used to produce nutritionally enriched cookies (high protein, ash and fiber content). In a study by Alka et al. [172], malt flour was added to the cookies, resulting in an increase in crude protein content, crude and dietary fiber and soluble fiber.

Since barley improves the nutritional properties and color of the cake, its use in the production of nutritionally-enriched cake is justified. Sprouted barley flour can be added to the recipe to obtain a cake enriched with nutrients and improved physical properties (crumbly and soft texture). In addition to improving the nutritional properties of the cake, the addition of sprouted barley flour in proportions of 10–40% has a significant anti-staling effect [173].

In their study, Yaqoob et al. [174] investigated the effects of adding raw and sprouted barley flour mixed with wheat flour on cake quality. They found that cakes with the addition of sprouted barley flour had both advantages and disadvantages. They were softer, firmer and more nutritious, but had weaker sensory characteristics compared to cakes made from pure wheat flour or raw barley flour.

In the research by Nakov et al. [37], the influence of replacing wheat flour with naked barley flour on the quality of short-dough cookies and the production of cookies with improved nutritional value and functional properties was analyzed. The research results showed that the physicochemical properties of cookies change significantly with the addition of barley flour (the spread factor and hardness decreased, and the color became slightly darker). At the same time, the addition of barley flour had a positive effect on the nutritional and functional properties of the cookies (the content of dietary fiber, especially β-glucan, and the content of total phenols and antioxidant capacity increased).

### 5.2. Malted Barley-Based Food Products

Malt production is an important technological process for processing whole grains. During beer production, large quantities of by-products are produced after mashing, such as roots or brewers’ spent grain (BSG) (approximately 85% of the total by-products obtained) [175]. Malted grain (or by-product of malting) can be used to produce a nutritionally-enriched, functional product with increased levels of fiber, minerals, proteins or secondary plant compounds. The increasing interest in the use of malted grains (or by-products resulting from the malting process) as a functional ingredient in the production of various cereal-based products is confirmed by research on this topic [36,37,45,176].

The most common application of barley malt is the production of beer and other beverages based on barley malt. However, small amounts of malt (in the form of malt flour) are also used in the production of baked goods. This type of malt flour is also called diastatic malt flour. Diastatic malt flour is commonly used in small amounts as an additive to improve amylolytic activity in the dough of baked goods that require fermentation. Non-diastatic malt extract is typically used to improve color and flavor in cookies and may contribute to the sweetness of the product as it contains significant amounts of sugar [69,177,178]. 

The study by Jukić et al. [38] referred to the use of barley malt flour (BMF) in the production of sponge cake. The influence of the addition of BMF, together with the reduction of sucrose in the recipe on the quality characteristics of sponge cake, was analyzed. The results showed that adding BMF influenced the reduction of specific volume and the coloration of the sponge cake crumb, positively affected the sensory evaluation of the product (with the addition of 20% BMF) and improved nutritional and functional properties of the sponge cake (since BMF contains significant amounts of its own sugar, which can minimize the effects of the reduction of sucrose content in the sponge cake recipe).

In another study by Jukić et al. [36], it was found that different types of BMF (enzymatically active Pilsen, low-enzymatically active Amber and non-enzymatically active Black) could be successfully used to produce functional sponge cake with reduced added sucrose. Furthermore, based on the results of sensory analysis, it was found that cookies with the addition of BMF (Pilsen and Amber) had a pleasantly sweet and rich taste. In contrast, the addition of Black BMF resulted in an excessively bitter taste and a nutty roasted aroma.

The use of barley malt roots as a by-product in the production of malt and beer contributes to the nutrient enrichment of the final product with proteins, essential amino acids, healthy fats, polyphenols and minerals. Waters et al. [176] investigated the possibility of producing nutrient-enriched bread by adding barley malt roots (fermented or just ground) in amounts up to 20%. The addition of barley malt roots in bread making can enrich bread and contribute to developing high-quality bread with improved characteristics for a wide consumer market.

BSG is the main by-product of the brewing industry, with a high content of proteins (20%) and fibers (70%). It contains about 17% cellulose and 28% non-cellulosic polysaccharides (mainly arabinoxylans and 28% lignin). Due to its granular structure, it must be milled into flour so that it can be added directly to the product. However, due to its influence on the color, flavor and texture of the product, there are certain limitations to the use of BSG as a flour, protein additive or a partial substitute for wheat flours. Because of its influence on the organoleptic and functional properties of the product, it is recommended to add BSG in a proportion of 5–10%. According to previous studies, BSG has been successfully used in a number of cereal-based products (bread, muffins, cookies, waffles, pancakes, tortillas, donuts, brownies and cakes) for the production of flakes and appetizers [45,179,180,181,182].

## 6. Conclusions

Consumers are becoming increasingly aware of the link between diet and disease. As a result, more attention is being paid to a balanced diet and more effort is now being put into developing novel, healthier, more nutritious and fortified functional foods worldwide.

Barley is an important source of macro- and micronutrients needed in the typical human diet, and has beneficial effects against the development of various diseases. Therefore, barley grain is gaining renewed attention worldwide due to its richness in functional ingredients (proteins, fibre, vitamins, and natural bioactive antioxidants such as phenols and lipids). These components are widely distributed in barley and influence the aroma, taste and color of foods. The nutritional properties of barley contribute to the prevention of numerous metabolic disorders by exerting antioxidant, anticarcinogenic, anti-inflammatory and cardio- and neuroprotective effects. Overall, the consumption of barley in the human diet has shown beneficial effects in preventing the development of chronic diseases. The recent interest in barley grain is mainly due to its content of soluble dietary fibre, β-glucan and antioxidant phytochemicals. However, studies on the bioactive and nutritional properties of barley and the use of the crop as a functional food in human nutrition are still limited.

Recently, there have been several initiatives to encourage barley producers to grow selected barley varieties suitable for human consumption and to develop barley-based products. Those barley varieties mainly include naked barley varieties and those that contain more β-glucan, higher levels of indigestible carbohydrates and better protein quality. Naked barley varieties require minimal processing because the bran layer remains intact, whereas covered varieties can lose nutrient-rich layers during pearling. In addition, cereal product manufacturers face numerous challenges as the addition of barley has an unfavorable effect on certain bakery products (reduced volume and increased water retention in the final product). Even though the addition of barley has a negative effect on some products, such as leavened bread, there are many products where the use of barley is very successful (e.g., flatbread, muffins, cookies).

## Figures and Tables

**Table 1:** Various forms of barley on the market.

Barley Type	Barley Form-Whole Grain	
Sub-types of covered barley	Covered Barley	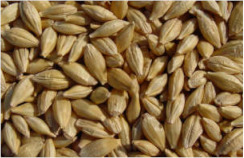
	De-hulled Barley	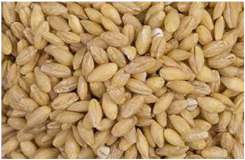
	Roasted Covered Barley	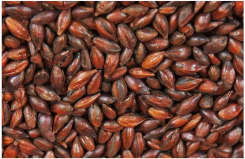
	De-hulled Barley Flakes	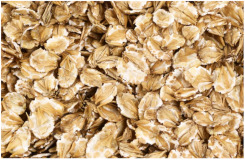
	Barley Malt 1. Pilsner malt 2. Pale Ale malt 3. Caramel malt 4. Chocolate malt 5. Roasted Barley malt	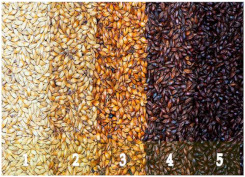
	Malted barley flour 1. Diastatic 2. Non-diastatic	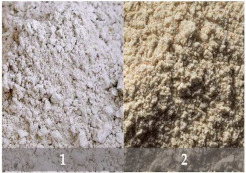
Sub-types of naked barley	Naked Barley	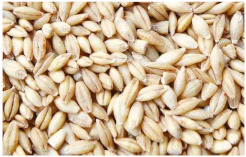
Sub-types of pearled barley	Pearled Barley	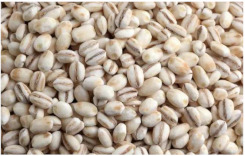
	Pot Barley	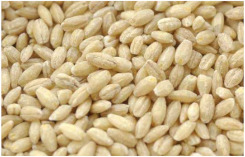
	Pearled Barley Flakes	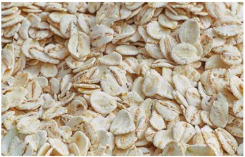
	Pearled Puffed Barley	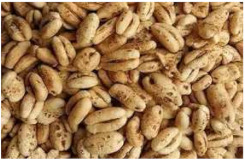
**Barley processing by-products**		
Sub-types of naked, covered and pearled barley	Barley Sprouts	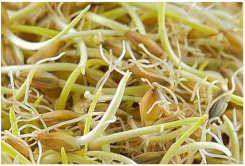
Sub-types of naked, covered and pearled barley	Barley Brewers’ Spent Grain	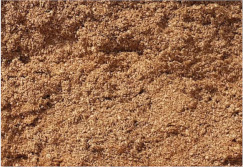
**Barley mill products**		
Sub-types of naked, covered and pearled barley	Barley Grits	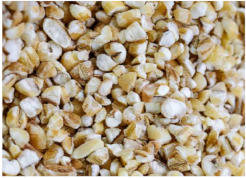
	Whole Grain Barley Flour	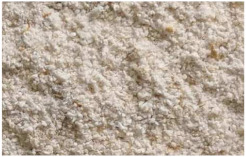
	Barley Bran	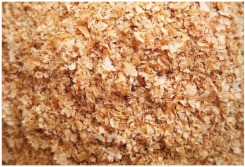
	Barley Husk	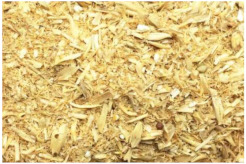
	Barley Starch	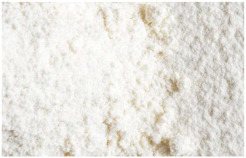

**Table 2:** Chemical composition of covered and naked barley grain [20].

Chemical Composition (%) *	Covered Barley	Naked Barley
Starch	57.7	60.7
Protein	12.2	15.1
Fat	2.5	2.7
Sugars	1.2	1.5
Ash	2.1	1.6
Dietary fiber	20.6	16.6
β-glucan	4.8	5.7

* Dry matter basis.

**Table 3:** The proportion of B-complex vitamins in barley (Adapted with permission from Ref. [9] 2022, John Wiley and Sons).

Vitamin	Average, mg/kg *
Thiamine (B_1_)	5.2
Riboflavin (B_2_)	1.8
Niacin (B_3_)	63.2
Pantothenic acid (B_5_)	5.1
Biotin (B_7_)	0.14
Folic acid (B_9_)	0.43
Pyridoxine (B_6_)	3.5
Choline	1290

* Dry matter basis.

**Table 4:** Barley-based traditional food prepared from whole, cracked or ground raw grain.

Food Category	Traditional Product Name	Reference
Food prepared from whole, cracked or ground raw grain	Balady, Pita, or Pocket Bread (Jordanian flatbread)	[99]
	Barley Porridge (Denmark porridge)	[21]
	Bolon or Boulon (Hard barley bread)	[99]
	Champa (Thin leavened pancake-like bread from Nepal)	[21,22]
	Chang (Tibetan alcoholic beverage)	[100]
	Chapatti (Indian flatbread)	[101]
	Chhaang or Chang (Nepalese and Tibetan alcoholic beverage)	[102]
	Dabbo (Ethiopian leavened thick bread)	[103]
	Drylur (Norway barley bread)	[98]
	Fateera or Kaac (Yemeni hard pie made of barley, corn and lentil flour)	[100]
	Fric and Mermez (Tunisian barley soup)	[104]
	Injera or Taita (Ethiopian and Eritrea thin leavened bread)	[105,106]
	Kaac (Yemen pie made from barley and lentil flour)	[100]
	Kicha (Flatbread from Eritrea)	[106]
	Kisra (Tunisian barley bread)	[104]
	Kitta or Torosho (Ethiopian unleavened, thin, dehydrated bread)	[103]
	Kornmjolsbrod (Swedish flatbread)	[22]
	Maloog and Matany (Yemen bread from barley and lentil flour)	[100]
	Malthouth (Tunisian dampen cracked barley couscous)	[104]
	Murri and Kamakh (Fermented condiment made with barley flour in Andalusia)	[98]
	Nakia (beverage from Yemen)	[21]
	Orkney Bere Bannocks (Scottish flatbread)	[22]
	Parathas and Poori (Indian single-layered flatbread)	[22]
	Paximadia (twice-baked barley biscuit)	[107]
	Sanchak tukba (Tibetan porridge)	[21]
	Shirba or Geat (Eritrean porridge)	[22]
	Shorba (Ethiopian soup)	[21]
	Tbikha (Tunisian cooked barley grain)	[104]
	Tihilo (Eritrean flour dough balls)	[108]
	Tortillas	[109]
	Toughrift (flatbread from Morocco)	[21]
	Turkey Tarhana, Egyptian Kishk, Iraqi Kushuk, Hungary Tahonya, Finland Talkuna	[110,111]
	Yufka (Turkish flatbread)	[112]
	Zoam, Matiat and Alaath (porridge from Yemen)	[22]
Food prepared from soaked, drained and dried whole or pearled grain	Ohralehikaiset (Finnish barley baked on leaves)	[21]
	Barley–Yogurt Soup	[9]
	Černy Kuba or Black Kuba (Czech mushroom–barley casserole)	[9]
	Goose and Barley Soup (Russian dish)	[113]
	Jau nu pani (barley water)	[108,114]
	Kasha (traditional sweet or savory dish of Russia, Poland and Eastern Europe)	[22]
	Kong Bori Bob (Korean cooked barley)	[114]
	Krupnick Polski (Polish barley–mushroom soup)	[9,115]
	Latvian Barley and Potato Porridge	[116]
	Latvian Bean Soup	[116]
	Miežu Putra or Meat and Barley Casserole (Latvian meat and barley casserole)	[9]
	Mugi Gohan (Japanese cooked barley)	[117]
	Polsa (Swedish barley sausage)	[22]
	Sanchak tukba (Tibetan porridge from steeped barley gain)	[100]
	Scotch Broth (Scottish soup)	[21]
	Talkkuna (in Finnish), Kama (in Estonian), Tolokno (in Russia) (powder which is a mixture of roasted barley)	[9]
Food prepared from malt, roasted grain or flour	Areki (Ethiopian distilled homemade drink from fermented ingredients)	[106]
	Atmit or Muk (Ethiopian soup from roasted grain flour)	[21]
	Bazine (Tunisian porridge from roasted grain flour)	[21,22]
	Besso and Chiko (Ethiopian dough balls with water, butter or oil)	[108]
	Boricha (Korean barley tea), Maicha (Chinese barley tea); Mugicha (Japanese barley tea)	[21,22]
	Buza	[98]
	Changuel and Tsangtub (Tibetan soup)	[21,100]
	Chima (Tibetan cake mixed with butter, dried cheese and sugar)	[21,100]
	Dhido (Etiopian porridge from roasted grain flour)	[21]
	Genfo or Kinche (Ethiopian porridge from roasted grain flour)	[21,106]
	Ini (fried naked barley grain)	[98]
	Kolo (Ethiopian and Eritrean mix with well-roasted whole grains served as breakfast, sidedish or snacks)	[106]
	Krimnitas or Chondrinos	[6,98]
	Magsan and Tsog (Tibetan cake mixed with tea, dried cheese, dried grapes and brown sugar)	[21,100]
	Pinni or Bagpinni (Himachal Pradesh snack from roasted barley flour)	[118,119]
	Sanchang (Tibetan slightly alcoholic beverage from roasted grain flour)	[100]
	Siwa (beer)	[106]
	Tella (Ethiopian fermented and undistilled beer)	[9]
	Tijmirout, Tounjifine and Tiroufine (Moroccan popcorn-like products)	[22]
	Tsangpa-ba or Tsampa-ba (Tibetan and Nepal dough balls with butter and tea)	[9,120]
	Yue (Tibetan popcorn-like snack)	[9]
	Yuetub (Tibetan porridge from roasted grain flour)	[21,22]
	Zurbegonie, Borde and Bequre (Ethiopian unfermented or slightly fermented beverage from roasted grain and malt)	[9,98]
